# A cluster of inappropriate shocks in a pediatric S-ICD patient - how to troubleshoot?

**DOI:** 10.1016/j.ipej.2023.12.006

**Published:** 2023-12-28

**Authors:** Christina Menexi, Mohamed ElRefai, David Farwell, Neil Srinivasan

**Affiliations:** a Cardiothoracic Center, Essex, UK; b Cambridge University Hospital, Cambridge, UK; c Circulatory Health Research Group, Medical Technology Research Centre, School of Medicine, Anglia Ruskin University, Essex, UK

**Keywords:** Subcutaneous implantable cardioverter defibrillator, Pediatric cardiology, Sudden cardiac death, Personalized device therapy, Inappropriate shocks

## Abstract

We present the case of a 16-year-old male pediatric patient diagnosed with hypertrophic cardiomyopathy (HCM, identified as having a high risk of sudden cardiac death (SCD), who underwent a successful subcutaneous implantable cardiac defibrillator (S-ICD) implantation as a primary prevention measure in 2018.

His past medical history included ADHD, Autism, and panic attacks. The patient experienced appropriate shocks that successfully terminated VF episodes. However, he also experienced multiple inappropriate shocks from the S-ICD, triggered by anxiety-induced tachycardia during panic episodes. Meticulous assessment of S-ICD tracings and electrocardiograms (ECGs) revealed the erroneous classification of sinus tachycardia as sustained ventricular tachycardia, leading to unwarranted therapeutic interventions.

Clinical intervention involved reprogramming of the S-ICD, emphasizing the pivotal role of personalized device configuration in pediatric cases where fine margins matter. While literature on S-ICD use in pediatric populations remains limited, emerging registries underscore the efficacy and safety of S-ICDs in preventing sudden cardiac death while reducing complications associated with intravascular leads. This case underscores the critical nature of customized device programming in pediatric patients, underscoring S-ICDs as a practical defibrillation alternative that addresses distinct concerns within this cohort of patients.

## 1 Case report

A 16-year-old male presented with known hypertrophic cardiomyopathy (HCM) due to a confirmed MYH7 mutation. He was initially diagnosed after presenting with chest pain and shortness of breath on exertion at the age of 9 years old. He had an S-ICD implanted in 2018 for primary prevention of sudden cardiac death (SCD). He was perceived as high risk of SCD due to his underlying cardiomyopathy and previous episodes of syncope concerning for underlying arrhythmia The patient also had a confirmed diagnosis of ADHD, Autism, and multiple panic attacks. His most recent echocardiogram in May 2023 indicated impaired left ventricular systolic function (EF 45 %), HCM, but no evidence of left ventricular outflow tract obstruction (LVOTO). Prior to admission, he was on Bisoprolol 2.5 mg OD and Lisinopril 10 mg OD. He was a non-smoker and did not consume alcohol.

He had a recent hospital admission due to two shocks from his S-ICD. Device interrogation identified a ventricular fibrillation (VF) arrest episode terminated by a successful shock, followed by another shock for wide complex tachycardia that failed to terminate the tachycardia. Initial Bisoprolol dose adjustment to 5 mg OD led to symptomatic bradycardia (dizziness). There was no documentation of pauses that would suggest sinus node disease. Regardless, Bisoprolol was reduced back to 2.5 mg OD. The patient and family chose to self-discharge against medical advice while awaiting further investigations and management.

**Fig. 1:**
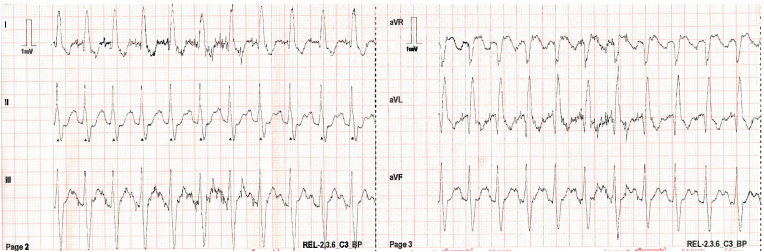

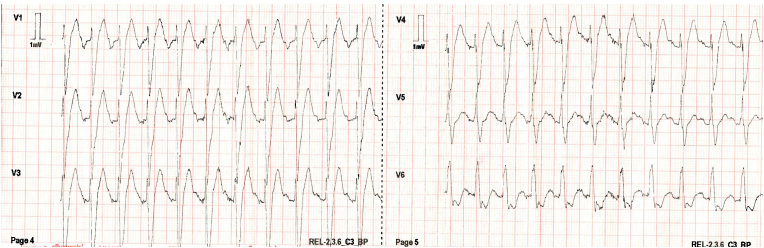
Ambulance 12-lead ECG showing sinus tachycardia at a rate of 150 beats per minute with wide QRS (140 msec).

Ten days post-discharge, the patient experienced sudden loss of consciousness witnessed by his family, with no preceding symptoms. Emergency services were contacted, and by the time they arrived, the patient was conscious and asymptomatic. An ambulance 12-lead ECG showed sinus tachycardia at 150 bpm with a QRS duration of 140 msec, see Fig. 1. Blood pressure was stable, and physical examination was unremarkable.

Initial blood tests, including serum electrolytes were unremarkable (Na 140 mEq/L, K 3.9 mEq/L, and Mg 0.92 mEq/L), his high sensitivity Troponin was only mildly elevated at 28ng/L (normal <14ng/L). The patient was admitted for further evaluation. While in the pediatric assessment unit, he became pale, dizzy, and anxious. Medical staff witnessed the patient receiving presumed multiple shocks from his S-ICD. The cardiac monitor indicated a heart rate of around 200 bpm. The patient remained conscious throughout. A preliminary diagnosis of ventricular tachycardia (VT) storm with multiple S-ICD shocks was made. He was administered 300 mg IV amiodarone, and urgent device interrogation was arranged. The shocks proved ineffective in terminating the arrhythmia, which eventually subsided without further shocks.

**Fig. 2:**
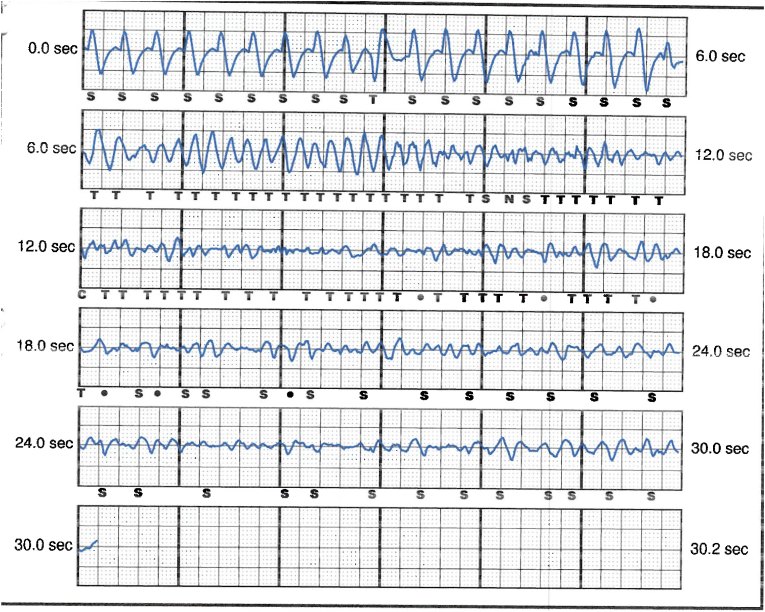

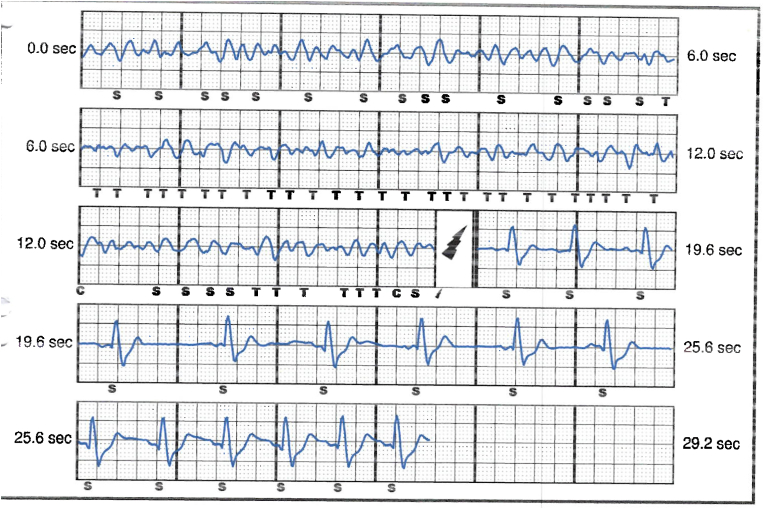
S-ICD interrogation revealed VF episode adequately identified and treated by the S-ICD.

**Fig. 3:**
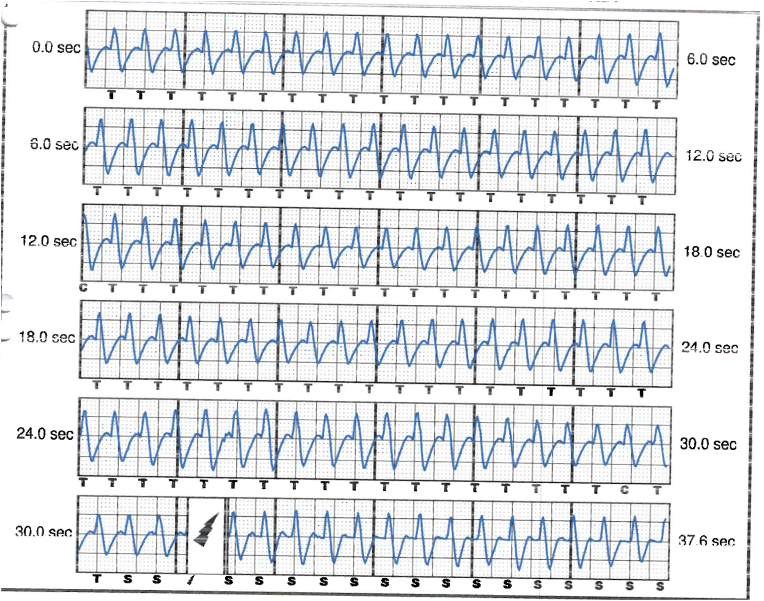

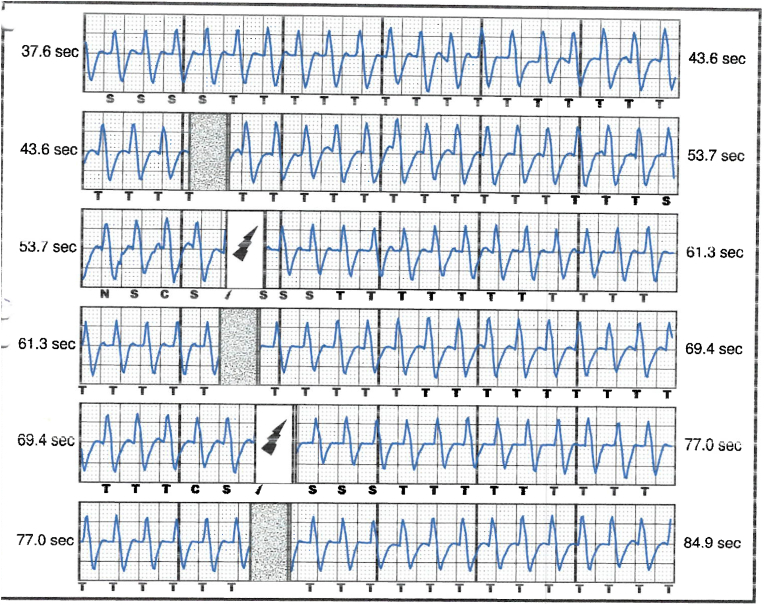

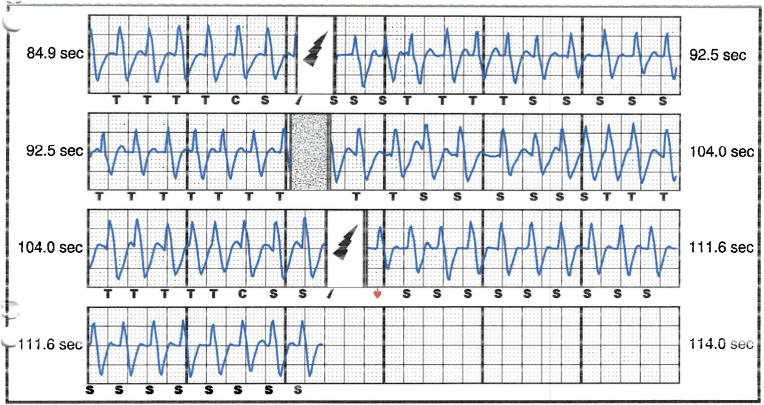

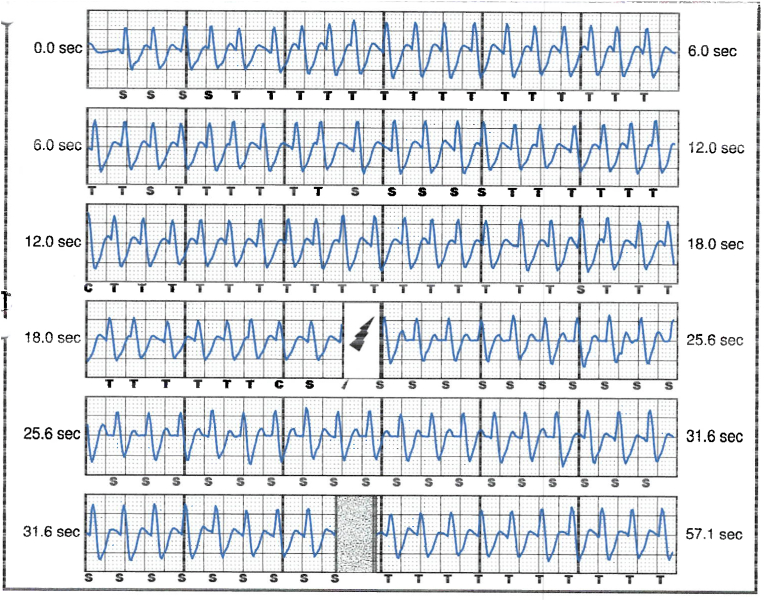

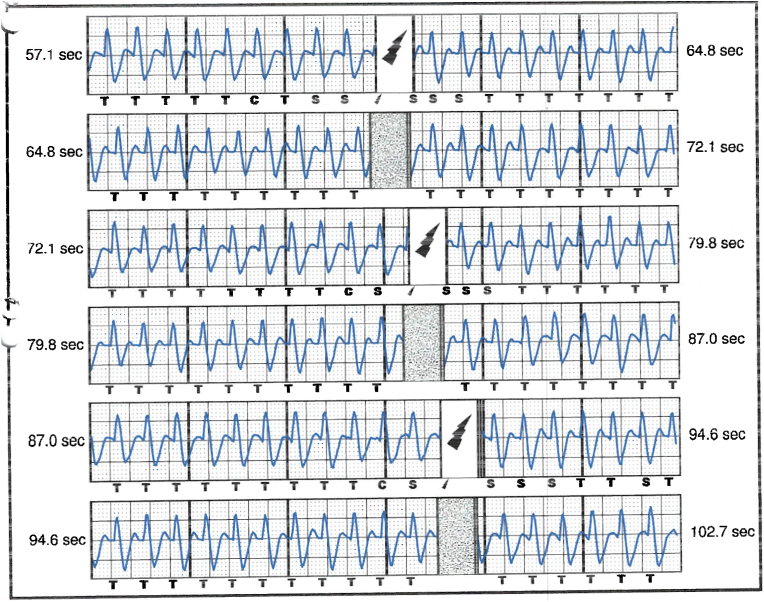

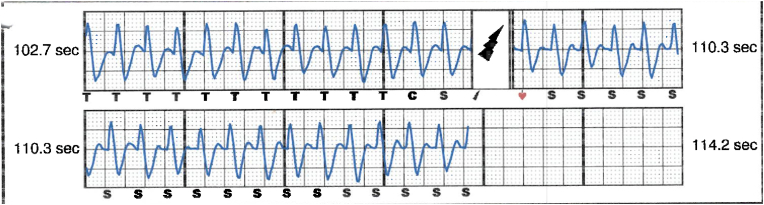

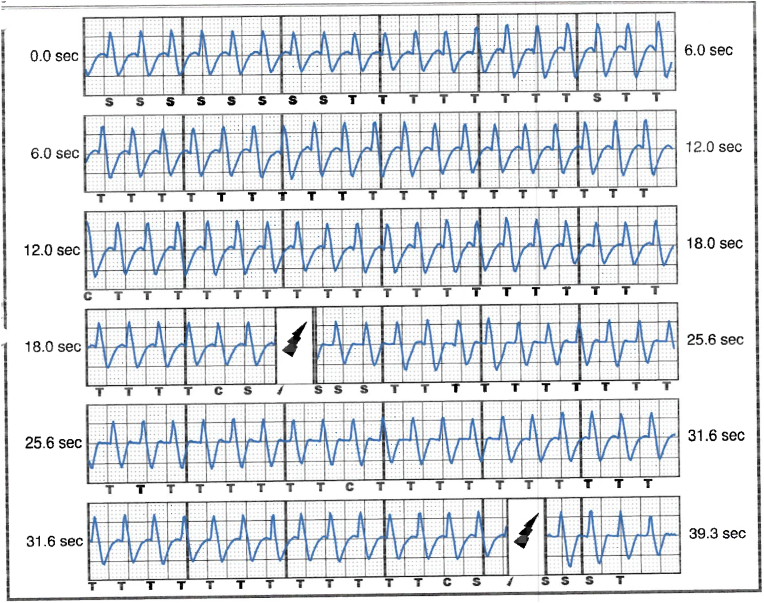

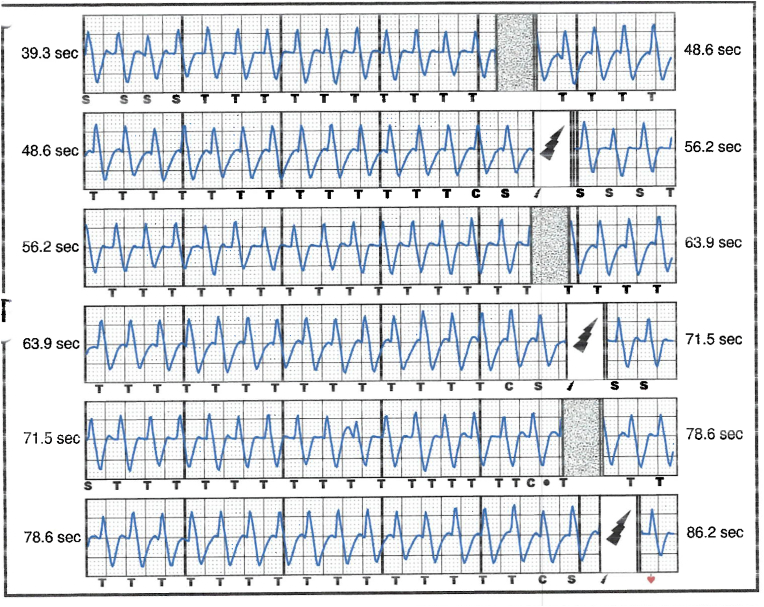

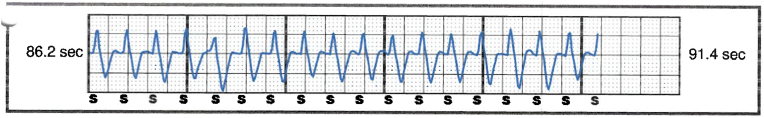
S-ICD tracings showing multiple shock therapies delivered by the S-ICD in response to sustained wide complex tachycardia at a rate of 200 bpm, inadequately identified as VT. The shocks failed to terminate the tachycardia.

S-ICD interrogation revealed evidence of VF effectively terminated by the device, 19 seconds into the episode which is considered satisfactory for S-ICD standards, during the initial loss of consciousness episode. Additionally, a total of 15 shock therapies were delivered by the S-ICD in response to sustained wide complex tachycardia at a rate of 200 bpm, identified as VT. The shocks had no appreciable effect on the presenting tachycardia and no further episodes were stored, see Fig. 2, Fig. 3. Device interrogation also revealed that the device was programmed to a conditional zone at 200 beats per minute and shock zone set at 220 beats per minute, the secondary vector was initially the programmed vector for sensing. S-ICD therapies were temporarily programmed off for further assessment.

**Fig. 4:**
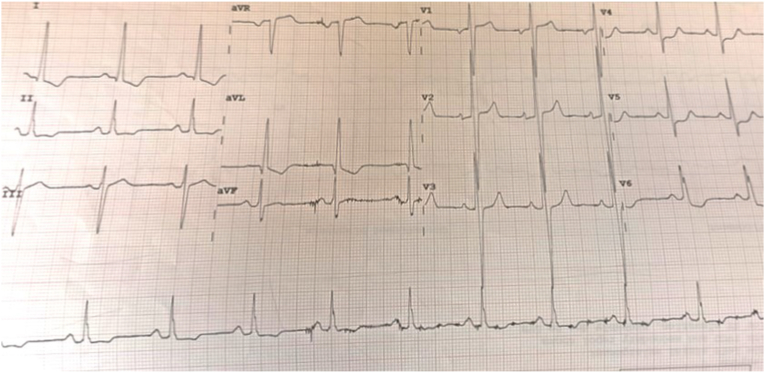
ECG in between panic attacks showing sinus rhythm with normal QRS duration (112 msec).

**Fig. 5:**
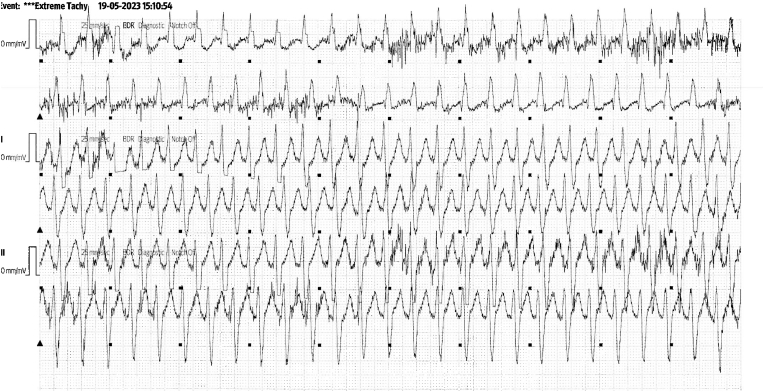
ECG tracing during panic attack showing sinus tachycardia with aberrancy.

**Fig. 6:**
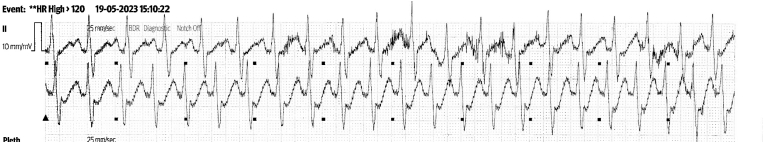
Shows tracing from the bedside monitor showing the gradual onset tachycardia in keeping with sinus tachycardia.

The patient was referred to electrophysiology services and admitted for further care. He received a full dose of 900 mg IV amiodarone for presumed VT storm. His resting heart rate was 50 bpm during sinus bradycardia, with evidence of gradual onset and offset of tachycardia (HR 150–170) during anxiety attacks, see Fig. 4, Fig. 5, Fig. 6. QRS widening during tachycardia indicated rate-related aberrancy. A thorough analysis of S-ICD traces suggested that the episode identified as persistent VT by the device was more consistent with sinus tachycardia with inadequately identified and treated aberrancy.

In view of his impaired LV function on the echocardiogram, a cardiac MRI was attempted to rule out underlying inflammatory cardiomyopathy. However, there was a significant artefact from his S-ICD, and the patient didn't tolerate the MRI well and it had to be abandoned without performing a full study, which was appreciated as an inconclusive study.

The patient and family were informed of the diagnosis. Amiodarone was discontinued, and Bisoprolol was switched to Sotalol 40 mg BD, titrated up to 80 mg BD to minimize further VF episodes. The S-ICD was reprogrammed to a conditional zone of 220 bpm and a shock zone of 250 bpm. After 72 hours of inpatient monitoring, the patient was safely discharged. Subsequent routine outpatient device interrogation was unremarkable, and the patient continues under regular clinical follow-up.

## 2 Discussion

### 2.1 S-ICDs

The subcutaneous implantable cardiac defibrillator (S-ICD) was designed to avoid complications of the transvenous implantable cardiac defibrillator (TV-ICD) by utilizing a totally extravascular approach. It comprises an electrically active can and a single subcutaneous lead containing two sensing electrodes: primary and distal. During implantation, the proximal electrode is sited 1cm inferior to the xiphisternum, fixated to the underlying muscle. The distal electrode is tunnelled to its final location, 14cm superior to the proximal electrode. The electrically active can creates a third sensing point. The S-ICD senses ECG signals and by measuring the voltage differences between these sensing points, it creates three different sensing vectors: primary, secondary, and alternate.

#### 2.1.1 Mechanism of action

**Fig. 7:**
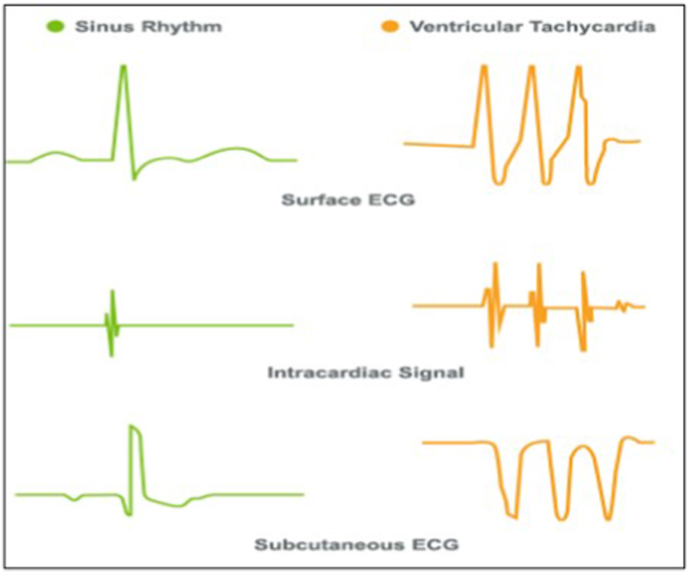
Surface ECG, intracardiac signal and S-ICD sensing vectors. Image © Boston Scientific Corporation or its affiliates. Reproduced with permission.

The vectors sensed by the S-ICD strongly resemble a surface ECG and the individual ECG components can be easily visually identified. This is different from the electrograms which are recorded in a TV-ICD system, see Fig. 7.

S-ICD treatment strategies are predominantly determined by heart rate. The heart rate is calculated by a continuous assessment of the selected vector amplitude using a pre-programmed sensitivity level. Amplitudes above the sensitivity level are identified as R waves whilst amplitudes below this level are ignored. The heart rate is calculated using the average of four consecutive R to R intervals. After each R wave detection, the S-ICD employs a blanking period, when no sensing occurs. This is to prevent double counting of a “wide” R wave with more than one peak, which can be seen in the presence of ventricular conduction disease.

The S-ICD therapies are programmable at two different therapy zones: the shock zone and the conditional shock zone. For the shock zone, the decision to deliver therapy is based solely on the heart rate. Once the heart rate exceeds the pre-programmed limit for the shock zone, shock therapy is delivered by the S-ICD. This is different for the conditional shock zone, which is usually set at a lower heart rate than the shock zone. Once the conditional shock zone is reached, the INSIGHT algorithm is activated. The INSIGHT algorithm uses 3 different discriminators (static morphology analysis, dynamic morphology analysis, and QRS width analysis) to differentiate between treatable and other high-rate events such as atrial fibrillation, sinus tachycardia and supraventricular tachycardia to help avoid inappropriate therapy. The Static morphology analysis Identifies non-shockable rhythms, utilizing the normal sinus rhythm template. The dynamic morphology analysis identifies shockable polymorphic rhythms by comparing each complex to the previous ones. While the QRS width analysis compares the QRS width to the normal sinus rhythm QRS width. If the INSIGHT algorithm identifies a sustained ventricular arrhythmia, shock therapy is delivered [1].

### 2.2 SICDs in the pediatric population

The use of S-ICD therapy in the pediatric population can be appealing as it eliminates the risks and complications that are associated with transvenous leads. However, the perceived higher rates of inappropriate shocks associated with S-ICDs can also make them less appealing. Children can easily achieve higher heart rates during their daily activities, and it is reasonable to speculate that higher heart rates can cause frequent activation of S-ICD detection systems which could increase the risk of inappropriate shocks. As such, there is a role in pre-SICD implant exercise screening to mitigate inappropriate sensing with exercise. This is common practice in many S-ICD implant centers.

There is less literature available on the use of S-ICDs in the pediatric population when compared to the adult S-ICD population. However, S-ICDs are now well-established therapy in the pediatric population. The overall outcome of the registries suggested that S-ICDs can effectively prevent sudden cardiac death in the pediatric population with a lower incidence of lead complications or device infections when compared to the TV-ICD systems [[2], [3], [4]].

### 2.3 Present case

The patient in this case is a young individual with strong indications for defibrillation protection. He is set to benefit from the lack of lead-related complications and lower risk of infective endocarditis associated with S-ICDs in contrast to the TV-ICD. He had multiple episodes of ventricular fibrillation (VF) arrests, effectively treated with his S-ICD. He has no clear modifiable risks for ventricular arrhythmias. He was not receiving any medications for treatment of his ADHD or panic attacks that would have potentially caused drug to drug interactions that would have precipitated the arrhythmias.

In addition to his cardiac condition, the patient had a diagnosis of Autism and a history of multiple anxiety attacks, during which his heart rate could also reach up to 200 beats per minute. The patient's young age (16 years old) was taken into account when predicting his maximum heart rate, which can reach up to 200 beats per minute based on age-adjusted equations. One equation that is commonly used to predict the maximum heart is the “208 - (0.7 × age)” equation proposed by Tanaka et al., which was developed in a meta-analysis study, after collecting data from the literature on 18,712 subjects [5]. Machado et al. tested the validity of this equation on a group of 69 apparently healthy children and adolescents (age 12.6 ± 1.5 years) via comparing the highest heart rate achieved during a maximal exertion test (HRmax) to the values predicted via the equation. there was no significant difference (p > 0.05) between the values predicted by the equation and the measured values. The authors concluded that the equation can be considered a valid for the examined age range. They also concluded that the constant value of 200 bpm can become the most appropriate value for HRmax in this population [6].

The S-ICD in this case was initially programmed with a conditional zone set at 200 beats per minute, and evidence of QRS prolongation at higher heart rates led to the perception of sinus tachycardia with aberrancy as a sustained VT, causing the delivery of inappropriate shock therapies. With each shock delivered, the pain and anxiety-driven tachycardia persisted, leading to a total of 15 inappropriate shocks in succession, before the heart rate slowed down gradually and subsequently no further shocks were delivered by the device. The reason why the conditional shock zone was kept at 200 bmp, which has proven to be low in our young patient, was not identified as his initial implant and programming was done under the care of another health care provider.

Interpretation of the S-ICD tracings and presenting ECGs (Fig. 2, Fig. 3, Fig. 5, Fig. 6) is more in keeping with a supraventricular origin of the tachycardia rather than a ventricular tachycardia, rendering the multiple shocks inappropriate. It is important to note that the S-ICD only stores relevant tachycardia events that surpass a certain threshold, as this helps save the limited device memory. This means that episodes of tachycardia that are not meeting the pre-programmed threshold would not be stored and thus not available for interpretation. Unfortunately, this means that warming up and cooling down traces of the clinical tachycardia are sometimes, as in our case, not available for interpretation. While there are some appreciable variations of R–R interval and morphology of QRS and we can't 100 % rule out the possibility of intermittent atrial arrhythmia, the presenting tachycardia is more in keeping with sinus tachycardia (with possible sinus arrhythmia) rather than atrial arrhythmia due to the gradual onset and offset perceived on the surface ECGs (see Fig. 5, Fig. 6), and the resistance to multiple shocks.

The management options were discussed with the patient and his parents, highlighting the need for defibrillation protection despite the inappropriate shocks. Two management options were presented: reprogramming the S-ICD parameters or switching to a transvenous ICD (TV-ICD) system, acknowledging the potential long-term complications associated with the transvenous approach and noting that inappropriate shock therapy via the transvenous ICD system is not uncommon. The decision was made to re-program the S-ICD to mitigate the risks of inappropriate sensing and further inappropriate shocks.

#### 2.3.1 Optimising the S-ICD programming

The vector that was programmed for sensing before the occurrence of the inappropriate shocks was the secondary vector. While in some cases of inappropriate shock therapy, changing the primary sensing vector can make a difference in reducing inappropriate shocks, we believe that changing the primary sensing vector to another (acceptable) sensing vector, without changing the threshold heart rate, would have had a limited effect on the inappropriate shocks in our case. There were demonstrable changes in the QRS durations in the surface ECG tracings during the tachycardia, see Fig. 5, which would have also most likely reflected on the signals sensed by all the vectors of the SICD, which undermines the chances of beneficial outcome of re-programming the device into a different sensing vector.

There is a role for pre-implant S-ICD exercise screening, which also might have an important role in post implant device re-programming particularly in the context of inappropriate shocks. However, our index patient has had his pre-implant screenings and implant procedure done at another facility and we have no evidence to suggest that he has had pre-procedure treadmill testing. Furthermore, we have not attempted exercise testing to guide our re-programming. In our index patient, the inappropriate sensing was triggered by the subtle changes in the QRS morphology at an inappropriately pre-set low heart rate threshold. We believe that by setting the threshold heart rates higher than the expected maximum physiological heart rate for age, we would tackle the main reason for the inappropriate sensing, not warranting exercise testing at this stage.

Following a shared decision-making process, the S-ICD was re-programmed with a conditional shock zone set at 220 beats per minute and a shock zone at 250 beats per minute, and the secondary vector was kept as the programmed sensing vector. If inappropriate sensing remains an issue, exercise testing remains a valid option to guide any further necessary re-programming changes.

The patient was closely monitored for a few days before being safely discharged from the hospital. Further follow-up at the device clinic did not reveal any evidence of additional appropriate or inappropriate therapy delivery, demonstrating the success of the chosen management approach.

This case highlights that special considerations are needed for the implantation as well as setting the S-ICD parameters in the pediatric patients’ cohort. It also emphasises on the importance of careful patient-specific device programming. As in our case, the finest of margins made all the difference.

## Ethical considerations

Informed written consent was obtained from the patient as well as from the parent/legal guardian for the publication of this case report. The consent forms specifically allowed for the use of anonymized clinical data and images for scientific and educational purposes.

## Declaration of competing interest

The authors declare that they have no known competing financial interests or personal relationships that could have appeared to influence the work reported in this paper.
